# Human alveolar macrophages predominately express combined classical M1 and M2 surface markers in steady state

**DOI:** 10.1186/s12931-018-0777-0

**Published:** 2018-04-18

**Authors:** Elena Mitsi, Raphael Kamng’ona, Jamie Rylance, Carla Solórzano, J. Jesus Reiné, Henry C. Mwandumba, Daniela M. Ferreira, Kondwani C. Jambo

**Affiliations:** 1 Department of Clinical Sciences, Liverpool School of Tropical Medicine, Liverpool, UK; 2 Malawi Liverpool Wellcome Trust Clinical Research Programme, College of Medicine, P.O Box 30096, Chichiri, Blantyre, 3 Malawi

## Abstract

Alveolar macrophages (AM) are critical to the homeostasis of the inflammatory environment in the lung. Differential expression of surface markers classifies macrophages to either classically (M1) or alternatively activated (M2). We investigated the phenotype of human alveolar macrophages (AM) in adults living in two different geographical locations: UK and Malawi. We show that the majority of AM express high levels of M1 and M2 markers simultaneously, with the M1/M2 phenotype being stable in individuals from different geographical locations. The combined M1/M2 features confer to AM a hybrid phenotype, which does not fit the classic macrophage classification. This hybrid phenotype may confer to alveolar macrophages an ability to quickly switch between M1 or M2 associated functions allowing for appropriate responses to stimuli and tissue environment.

**Electronic supplementary material:**

The online version of this article (10.1186/s12931-018-0777-0) contains supplementary material, which is available to authorized users.

## Introduction

The healthy human alveoli are dominated by alveolar macrophages (AM) [1]. These cells play a critical role in regulating immune responses [2] and maintaining homeostasis in the lung [1, 3]. Their plasticity to adapt to changes in their microenvironment is fundamental in retaining the lung health [4]. Macrophages have been categorised as “polarised” towards either M1 (proinflammatory, mediating resistance to pathogens), or M2 mode (anti-inflammatory, promoting tissue remodelling) [5]. This categorisation is derived from murine models, and from in vitro polarisation of human macrophages using a combination of cytokines. However, whether AM polarisation in steady state fits the traditional M1/M2 dichotomy is still not well understood. We therefore investigated human AM polarisation in steady state, and ascertained the stability of the phenotype in two geographical locations and following in vivo exposure to bacterial and viral stimuli.

### Methods

Briefly, in the UK, healthy, non-smoking participants aged 18–50 years were recruited from an ongoing study of the Experimental human pneumococcal challenge (EHPC) model [6]. A subset of 25 participants, 16 individuals who had become colonized (carriage +) with *Streptococcus pneumoniae*, and 9 who had not (carriage –) underwent research bronchoscopy at 4 to 7 weeks after the bacterial challenge. Alveolar macrophages were obtained from bronchoalveolar lavage (BAL), as described previously [7]. In Queen Elizabeth Central Hospital (QECH), Malawi, BAL samples [8] were obtained from selected asymptomatic adults (≥18 yrs), comprising ten HIV-1-uninfected healthy controls and ten age-matched ART-naïve HIV-1-infected individuals. We excluded participants with clinical evidence of active disease or recent history of severe respiratory illness.

Flow cytometry-based immunophenotyping was used to characterise AM phenotype obtained from BAL fluid. The expressional levels of key surface markers were measured immediately after sample collection.

## Results

### Human alveolar macrophages simultaneously express M1 and M2 markers in steady state

**Fig. 1:**
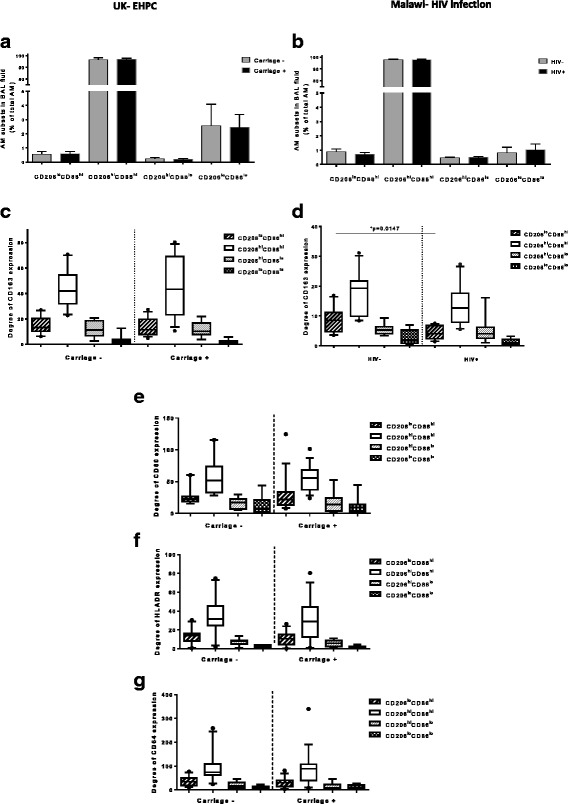
Comparison of human alveolar macrophage phenotype between UK and Malawi healthy adults (**a**) Percentages of AM subsets gated for CD206 and CD86 (UK cohort, *n* = 9 for grey bars and *n* = 16 for black bars) and the (**b**) the degree of CD163 expression in each of the subsets. The data shows that CD206^hi^CD86^hi^ subset occupies the highest proportion and expresses the highest levels of CD163 when compared with the other three. **c**-**d** The same pattern is observed on the AM derived from the healthy Malawian individuals (grey bars, *n* = 10). The effect of pneumococcal colonization and HIV infection on AM polarisation. **a**-**b** AM subsets are compared between non-colonized (carriage -) and colonized group (carriage +) post experimental nasal pneumococcal inoculation. There is no alteration of AM phenotype associated with nasal pneumococcal colonization. CD206^hi^CD86^hi^ is the dominant subset with the highest CD163 expression amongst the rest (*p* < 0.0001 when compared with CD206^lo^CD86^hi^ or CD206^hi^CD86^lo^. **c** AM subsets are compared between HIV-uninfected healthy adults (n = 10) and HIV-infected individuals (*n* = 10). AM collected from HIV-infected individuals follows the same pattern with healthy HIV-uninfected individuals when gated for CD206 and CD86. **d** CD206^hi^CD86^hi^ is the dominant subset with the highest CD163 expression amongst the rest (*p* < 0.002 when compared with CD206^lo^CD86^hi^ or CD206^hi^CD86^lo^ in both HIV-uninfected and HIV-infected individuals. However, the HIV infection regresses the expression of CD163 in the CD206^lo^CD86^hi^ subset. **e**-**g** Levels of CD80, CD64 and HLADR expression respectively between carriage negative and carriage positive individuals. There is no significant difference on levels of CD80, CD64 and HLADR expression mediated by nasopharynx pneumococcal colonization. Within-group comparisons used Wilcoxon tests, and between-cohort comparisons used Mann-Whitney U test

Data from the two human cohorts from distinct geographical locations, UK (Additional file 1 Table S1) and Malawi (Additional file 1 Table S2), were collected independently and their comparison revealed a similar pattern of surface marker expression by AM. In both cohorts, the majority of AM were characterised as CD206^hi^CD86^hi^. M1-like phenotype (CD206^lo^CD86^hi^) and M2-like phenotype (CD206^hi^CD86^lo^) subsets represented a small proportion (less than 1%) of the total AM population (Fig. 1a and c). In addition, the CD206^hi^CD86^hi^ subset expressed greater levels of the M2 marker, CD163, compared to the M1 and M2 subpopulations (Fig. 1b and d). UK samples were further analysed for additional M1 markers (CD80, CD64) and an activation marker (HLADR). In these, the CD206^hi^CD86^hi^ subset expressed the highest levels of CD80, CD64 and HLADR expression in comparison to the other AM subsets (Fig. 1e-g).

### Colonization with pneumococcus does not alter the AM phenotype, whereas chronic HIV infection is associated with a lower expression of CD163 on CD206^lo^CD86^hi^ AM

We have previously shown that live intranasal pneumococcal challenge alters the alveolar environment by increasing the levels of pneumococcal-specific memory CD4^+^ Th17 cells in the human lung [9], and that HIV infection disrupts the alveolar cytokine microenvironment [10]. We therefore investigated whether in vivo exposure to intranasal pneumococcal challenge or HIV infection polarise the AM phenotype. We compared experimentally pneumococcal-colonized individuals with non-colonized individuals, as well as, asymptomatic ART-naïve HIV-infected adults with age-matched HIV-uninfected healthy individuals. We found no difference in M1 or M2-like phenotype between individuals who became colonized following inoculation with *S. pneumoniae* compared with those who cleared the bacteria (Fig. 1a). Specifically, the expression of CD163 (Fig. 1b), CD80, CD64 and HLADR measured in the four AM subsets remain at the same levels between these groups (Fig. 1e-g).

Interestingly, in the Malawian population, AM subsets from HIV-infected individuals followed the same pattern of subdivision described in healthy volunteers. No difference was found in the proportion of the investigated subsets between the ART-naïve HIV-infected adults and HIV-uninfected healthy individuals (Fig. 1c). However, the expression of CD163 was significantly reduced in the CD206^lo^CD86^hi^ subset in HIV-infected individuals (*p* = 0.0147) (Fig. 1d).

## Discussion

In our study, we compared the polarisation of AM isolated from human volunteers from distinct geographical locations, UK and Malawi. We report that in healthy human pulmonary mucosa, AM adopt a hybrid phenotype, which shares features of both M1 (CD80, CD86, CD64) and M2 (CD206 and CD163) polarized macrophages. The same phenotype has been previously reported on the decidual macrophages (resident macrophages of the uterine lining), which exhibit characteristics of both pro-inflammatory and tolerogenic macrophages [11].

We also found that nasal pneumococcal colonization, even though it alters CD4^+^ T cell responses in the alveoli [9], does not alter the expression of key M1 and M2 polarization markers on the surface of AM. This leads to the speculation that a nasal pneumococcal exposure episode is either incompetent to modulate AM polarization or its effect is transient and quickly irreversible due to macrophages high plasticity [4]. By contrast, chronic HIV infection did affect the expression of CD163 on the M1-like AM subset (CD206^lo^CD86^hi^), with a lower expression of CD163 observed in HIV-infected individuals than HIV-uninfected controls. This finding is intriguing and may be due to active HIV infection of this alveolar macrophage subset [12] which has been shown to strongly repress CD163 expression on infected-macrophages [13]. Furthermore, CD163 is shed during activation as soluble CD163 (sCD163) [14] and this might in part explain the reduction of CD163 on the surface M1-like AM in HIV-infected individuals. However, even with in vivo exposure to pneumococci or HIV, the CD206^hi^CD86^hi^ AM subset remained the major population, and this ascertains the stability of this phenotype.

Our data provides strong evidence suggesting that not all macrophages fall into M1 and M2 subsets. The major AM population in steady state expresses a duo M1/M2 phenotype. This phenotype is present in individuals from two distinct geographical location and is stable even after exposure to stimuli known to alter the alveolar environment. This is consistent with recent call for a rethink of the M1/M2 macrophage paradigm [5]. It is possible that the lack of stark polarisation is helpful in maintaining a healthy balance between immune tolerance and protective immunity in the alveolar space. However, whether other tissue macrophages, beyond decidual and alveolar macrophages, exhibit similar phenotypes in vivo warrants further investigation.

## Conclusion

The clear majority of alveolar macrophages combine M1 and M2 features in steady state, a phenotype that may allow brisk and adaptive responsiveness to multiple elements in the local milieu.

## Additional file

Additional file 1: **Table S1.** Demographics of the EHPC study participants- UK. **Table S2.** Demographics of study participants – Malawi. **Table S3.** Summary of the panel composition at the UK site (no shading) and Malawi site (grey shading). **Fig. S1.** Gating strategy used to identify human alveolar macrophages in the UK Cohort. **Fig. S2.** Gating strategy used to identify human alveolar macrophages in the Malawi Cohort. (DOCX 681 kb)

## Abbreviations

AM: Alveolar macrophage
BAL: Bronchoalveolar lavage
