# Supplementary material for: Human alveolar macrophages predominately express combined classical M1 and M2 surface markers in steady state
Source: Respir Res. 2018 Apr 18;19:66. doi: 10.1186/s12931-018-0777-0 (PMC5907303; doi:10.1186/s12931-018-0777-0)
Supplement: Supplementary file 1 — Table S1. Demographics of the EHPC study participants- UK. Table S2. Demographics of study participants – Malawi. Table S3. Summary of the panel composition at the UK site (no shading) and Malawi site (grey shading). Fig. S1. Gating strategy used to identify human alveolar macrophages in the UK Cohort. Fig. S2. Gating strategy used to identify human alveolar macrophages in the Malawi Cohort. (DOCX 681 kb) [file 12931_2018_777_MOESM1_ESM.docx]

**SUPPLEMENTARY MATERIAL**

| **UK Cohort** | **Pneumococcal non-colonized**  **(n=9)** | **Pneumococcal colonized**  **(n=16)** | **p value** |
| --- | --- | --- | --- |
| **Median age (range) -yr** | 20 (19-25) | 21 (18-31) | p>0.05 |
| **Sex (M:F)** | 4:5 | 7:9 |  |
| **Median inoculation dose (range) - *CFU/nostril** | \| 82,500 \| \| --- \|   (76667-89,000) | \| 87,500 \| \| --- \|   (77500-93,000) | p>0.05 |
| **Median duration of carriage (range)- days post inoculation** | N/A | 27.0 (14.0-27.0) |  |

*CFU= colony forming units

**Table S1: Demographics of the EHPC study participants- UK**

| **Malawi Cohort** | **HIV-uninfected**  **(n=10)** | **HIV-infected ART-naive (n=10)** | **p value** |
| --- | --- | --- | --- |
| **Median age (range) -yr** | 33 (20-35) | 33 (22-35) | p>0.05 |
| **Sex (M:F)** | 7:3 | 7:3 |  |
| **Peripheral blood CD4 count (cells/μl), median (IQR)** | 664 (469-746) | 251 (154-356) | 0.0001 |
| **White blood cell count (cells/L), median (IQR)** | 4.4 (3.4-4.8) | 4.7 (4.0-6.4) | 0.4692 |
| **Haemoglobin (g/dL, median (IQR)** | 15.6 (14.3-16.5) | 13.0 (11.3-13.8) | 0.0121 |
| **Haematocrit (%), median (IQR)** | 47.4(41.8-49.7) | 38.9(35.2-41.4) | 0.0140 |

**Table S2: Demographics of study participants – Malawi**

**METHODS**

**Experimental human pneumococcal intranasal challenge and carriage determination – UK cohort**

In the UK, healthy, non-smoking participants aged 18-50 years were recruited from an ongoing study of the Experimental human pneumococcal challenge (EHPC) model [1]. Experimental human pneumococcal challenge (EHPC) was conducted at Liverpool as previously described[1, 2]. Briefly, mid-log-growth vegitone culture of *Streptococcus pneumoniae* serotype 6B (strain BHN418) was prepared and stored at -80^o^C, and independently tested by Public Health England for purity and antibiotic sensitivity. 80,000 colony-forming-units (CFU) were sprayed into each nostril of participants, inoculated within 30 minutes of stock dilution. Dilution plating was used to confirm the dose received. Pneumococcal colonization was detected by classical microbiology methods and quantified by serial dilution from nasal washes performed at days 2,7,9 and 27 [1-3]. For pneumococcal colonized individuals, additional washes occurred at days 14 and 21. Serotype was confirmed by latex agglutination (Statens Serum Institute, Copenhagen, Denmark) [4]. Within a time-window of 4 to 7 weeks after the intranasal challenge, a subset of volunteers, 16 individuals who had become colonized (carriage+) with the pneumococcus and 9 who had not (carriage–) underwent research bronchoscopy in the Clinical Research Unit (CRU) at the Royal Liverpool University Hospital (RLUH) [5].

**Malawi Cohort- Study participants**

The study was conducted at the Queen Elizabeth Central Hospital, a large teaching hospital in Blantyre, Malawi. Participants were recruited from the hospital’s voluntary counselling and testing (VCT) and ART clinics. They were adults aged ≥18yrs comprising healthy HIV-1-uninfected and asymptomatic HIV-1-infected volunteers with no clinical evidence of active disease and willing to undergo bronchoscopy and BAL for research purposes [6]. HIV testing was performed on whole blood using two commercial point-of-care rapid HIV test kits, Determine HIV 1/2 kit (Abbott Diagnostic Division) and Unigold HIV 1/2 kit (Trinity Biotech Inc.). A participant was considered HIV-uninfected if the test was negative by both kits or HIV-infected if the test was positive by both kits. If Determine and Unigold results were discordant, a third rapid test using Bioline HIV 1/2 kit (Standard Diagnostics Inc.) was performed to resolve the discordance. None of the participants were on ART at the time of recruitment to the study, but all initiated ART after sample collection according to the 'test and treat' Malawi national treatment guidelines. Exclusion criteria for the study were: current or history of smoking, use of immunosuppressive drugs, severe anaemia (Hb<8g/dl) and known or suspected pregnancy.

**Sample collection and experimental procedures**

Bronchoscopy and BAL were performed on all participants as previously described for the UK [5] and Malawi cohort [7] respectively. The fluid was filtered using sterile gauze and centrifuged at 400g for 10 min at 4 °C. The supernatant was removed, the cell pellet was resuspended and washed with PBS. The centrifugation step was repeated once, and the cell pellet was resuspended in cold RPMI medium (Gibco™ RPMI 1640 Medium) containing antibiotics (Penicillin, Neomycin and Streptomycin, Sigma-Aldrich, Sigma Chemical Co). Cell counts in BAL cells from each sample were performed using a haemocytometer.

**Immunophenotyping**

Whole BAL cells (1 x 10^6^ cells) were stained with predetermined optimal concentration of flourochrome-conjugated monoclonal antibodies against human cell surface proteins. At the UK site, cells were stained with Aqua Viability dye (LIVE/DEAD® Fixable Dead Cell Stain kit, Invitrogen, UK), anti-CD45 FITC, anti-CD80 APC-H7, anti-CD86 PE, anti-CD206 PE-CF594, anti-CD14 PerCP Cy5.5, anti-CD16 PE Cy7, anti-CD163 APC, anti-CD11b AF700, anti-CD11C PB, anti-CD64 BV605 and anti-HLADR BV785. All the samples were acquired on the FacsAria III sorter/cytometer (BD Biosciences). At the Malawi site, the cells were stained with anti- Flow cytometry data were analysed using FlowJo software (TreeStar, USA). CD45 Alexa Fluor 700, anti-CD206 APC, anti-CD66b FITC, anti-CD163 Brilliant Violet 421, and anti-CD86 PE Dazzle (All Biolegend, UK). Further details of the antibodies are included in Table S3. Compensation was set using CompBeads (BD Biosciences). All samples were acquired on the BD LSRFortessa flow cytometer (BD Biosciences). Flow cytometry data were analysed using FlowJo software (TreeStar, USA) (Figure S1 and S2). Expression of surface markers was calculated by subtracting the Median Fluorescent Intensity (MFI) of the surface marker on the AM population from the Median Fluorescent Intensity (MFI) of the surface marker on the Neutrophil population (negative control), divided by twice the robust standard deviation of the MFI of the surface marker on the neutrophil population.

| **Marker** | **Fluorochrome** | **Clone** | **Provider** | **Filter** | **Isotype** |
| --- | --- | --- | --- | --- | --- |
| CD45 | FITC | HI30 | Biolegend | 530/30 | IgG1 |
| CD80 | APC-H7 | L307.4 | BD Biosciences | 780/60 | IgG1 |
| CD86 | PE | IT2.2 | Biolegend | 585/42 | IgG2b |
| CD206 | PE-CF594 | 19.2 | BD Biosciences | 616/23 | IgG1 |
| CD14 | PerCPCy5.5 | MƟP9 | BD Biosciences | 695/40 | IgG2b |
| CD16 | PECy7 | 3G8 | Biolegend | 780/60 | IgG1 |
| CD163 | APC | GHI/61 | Biolegend | 660/20 | IgG1 |
| CD11b | AF700 | ICRF44 | BD Biosciences | 730/45 | IgG1 |
| CD11c | PB | Bu15 | Biolegend | 450/50 | IgG1 |
| CD64 | BV605 | 10.1 | Biolegend | 610/20 | IgG1 |
| HLADR | BV785 | L243 | Biolegend | 780/60 | IgG2a |
| Live & Dead | BV510 | NA | Invitrogen | 510/50 | NA |
| CD206 | APC | 15-2 | Biolegend | 660/20 | IgG1 |
| CD66b | FITC | G10FG | Biolegend | 530/30 | IgM |
| CD163 | Bv421 | GHI/61 | Biolegend | 450/50 | IgG1 |
| CD45 | AF700 | 2D1 | Biolegend | 730/45 | IgG1 |
| CD86 | PE DAZZLE | IT2.2 | Biolegend | 616/23 | IgG2b |

**Table S3. Summary of the panel composition at the UK site (no shading) and Malawi site (grey shading)**.

### **Statistical analysis**

In within-group comparisons Wilcoxon tests were used, whereas between-different groups comparisons Mann-Whitney U test were used. Statistical analyses were performed with Graph Pad Prism version 6.0, Graph Pad Software, La Jolla, CA, USA).

**
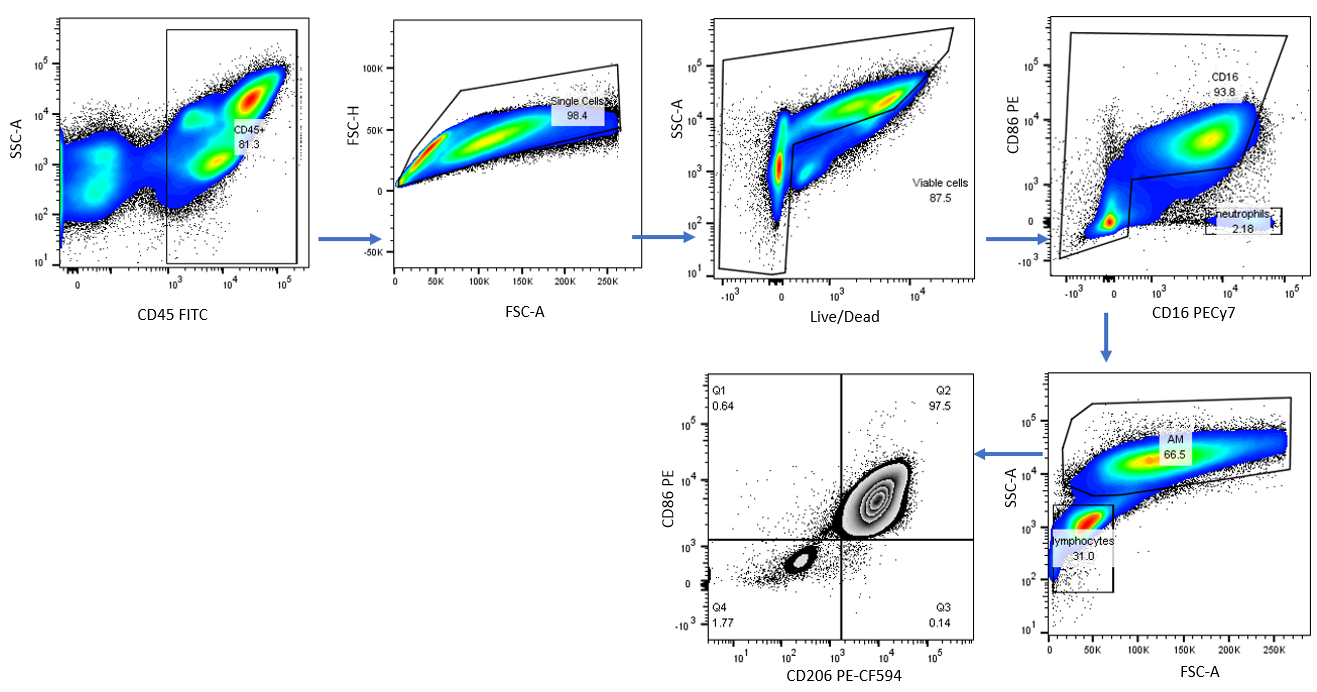
**

**Figure S1. Gating strategy used to identify human alveolar macrophages in the UK Cohort**

Leucocytes were identified as CD45+, following exclusion of doublets and dead cells (precaution was taken not to gate out highly autofluorescent alveolar macrophages). Neutrophils were identified as CD45+CD16+CD86- cells. Alveolar macrophage (AM) and lymphocyte populations were identified by their forward scatter (FSC) and side scatter (SSC) characteristic properties. Using CD206 and CD86 we separated AM to four subpopulations: CD206^lo^CD86^hi^, CD206^hi^CD86^hi^, CD206^hi^CD86^lo^ and CD206^lo^CD86^lo^. Neutrophils and lymphocytes were used as the negative population to calculate the degree of expression of AM surface markers CD163, HLA-DR, CD64 and CD80, respectively.


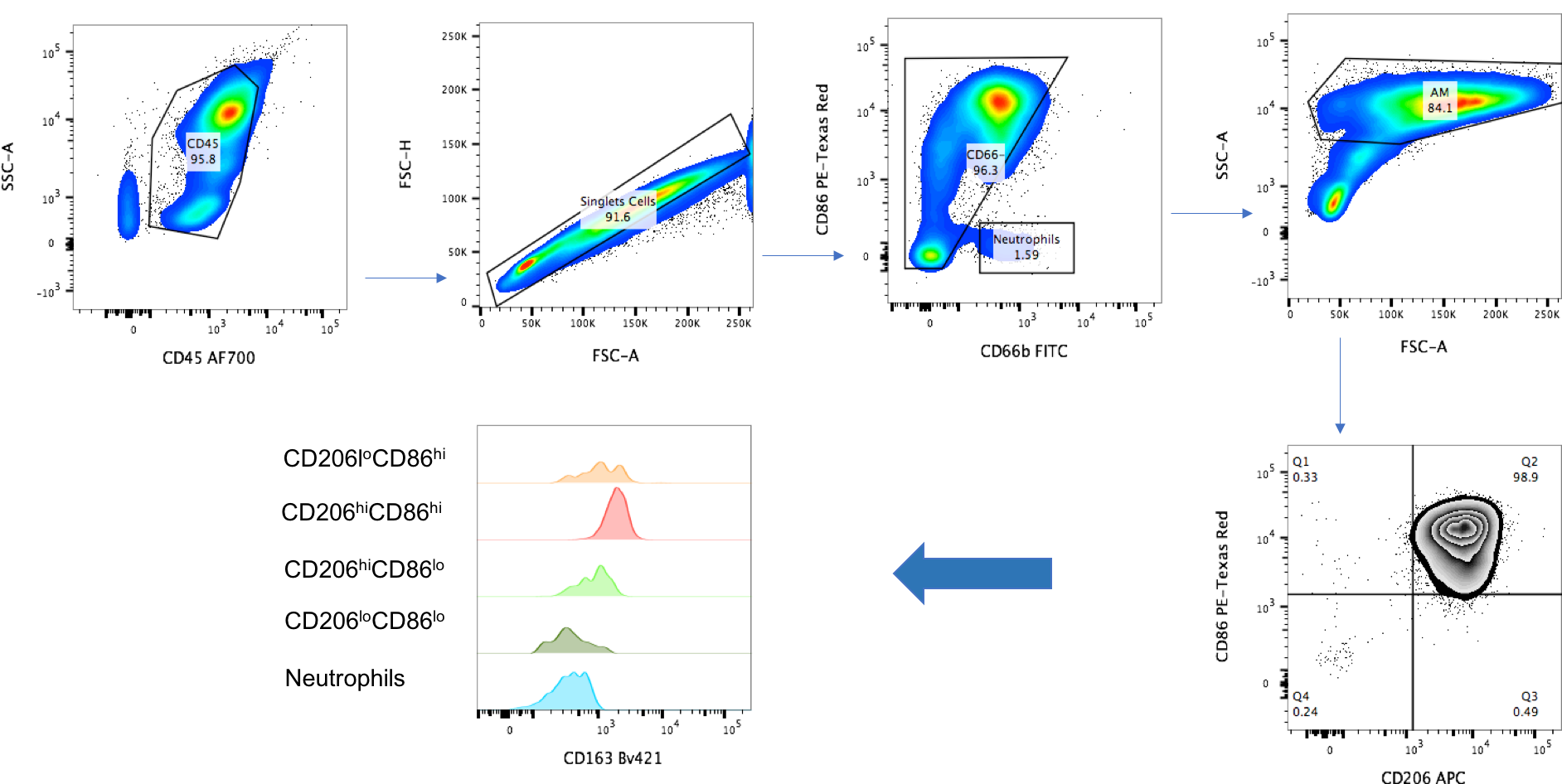


**Figure S2. Gating strategy used to identify human alveolar macrophages in the Malawi Cohort.**

The same gating strategy applied on the Malawi cohort, with a difference of a neutrophils marker. Here we used CD66b to identify neutrophils as CD45+CD66b+CD86-. AM gated as described above and the degree of CD163 expression on the surface of AM subsets calculated using neutrophils as the negative population.

**REFERENCES**

1. Gritzfeld JF, Wright AD, Collins AM, Pennington SH, Wright AK, Kadioglu A, Ferreira DM, Gordon SB: **Experimental human pneumococcal carriage**. *J Vis Exp* 2013(72).

2. Ferreira DM, Neill DR, Bangert M, Gritzfeld JF, Green N, Wright AK, Pennington SH, Bricio-Moreno L, Moreno AT, Miyaji EN *et al*: **Controlled human infection and rechallenge with Streptococcus pneumoniae reveals the protective efficacy of carriage in healthy adults**. *American journal of respiratory and critical care medicine* 2013, **187**(8):855-864.

3. Pennington SH, Pojar S, Mitsi E, Gritzfeld JF, Nikolaou E, Solorzano C, Owugha JT, Masood Q, Gordon MA, Wright AD *et al*: **Polysaccharide-Specific Memory B Cells Predict Protection against Experimental Human Pneumococcal Carriage**. *American journal of respiratory and critical care medicine* 2016, **194**(12):1523-1531.

4. Gritzfeld JF, Cremers AJ, Ferwerda G, Ferreira DM, Kadioglu A, Hermans PW, Gordon SB: **Density and duration of experimental human pneumococcal carriage**. *Clin Microbiol Infect* 2014, **20**(12):O1145-1151.

5. Zaidi SR, Collins AM, Mitsi E, Reine J, Davies K, Wright AD, Owugha J, Fitzgerald R, Ganguli A, Gordon SB *et al*: **Single use and conventional bronchoscopes for Broncho alveolar lavage (BAL) in research: a comparative study (NCT 02515591)**. *BMC Pulm Med* 2017, **17**(1):83.

6. Mtunthama N, Malamba R, French N, Molyneux ME, Zijlstra EE, Gordon SB: **Malawians permit research bronchoscopy due to perceived need for healthcare**. *J Med Ethics* 2008, **34**(4):303-307.

7. Jambo KC, Banda DH, Afran L, Kankwatira AM, Malamba RD, Allain TJ, Gordon SB, Heyderman RS, Russell DG, Mwandumba HC: **Asymptomatic HIV-infected individuals on antiretroviral therapy exhibit impaired lung CD4(+) T-cell responses to mycobacteria**. *American journal of respiratory and critical care medicine* 2014, **190**(8):938-947.
